# Acute Insulin Secretory Effects of a Classic Ketogenic Meal in Healthy Subjects: A Randomized Cross-Over Study

**DOI:** 10.3390/nu15051119

**Published:** 2023-02-23

**Authors:** Alberto Battezzati, Andrea Foppiani, Alessandro Leone, Ramona De Amicis, Angela Spadafranca, Andrea Mari, Simona Bertoli

**Affiliations:** 1 ICANS-DIS, Department of Food Environmental and Nutritional Sciences, University of Milan, 20133 Milan, Italy; 2 Clinical Nutrition Unit, Department of Endocrine and Metabolic Medicine, IRCCS Istituto Auxologico Italiano, 20100 Milan, Italy; 3 Obesity Unit and Laboratory of Nutrition and Obesity Research, Department of Endocrine and Metabolic Diseases, IRCCS Istituto Auxologico Italiano, 20145 Milan, Italy; 4 National Research Council (CNR), Institute of Neuroscience, 35127 Padua, Italy

**Keywords:** ketogenic diet, Mediterranean diet, insulin secretion

## Abstract

The classic ketogenic diet (KD) is a high-fat, low-carbohydrate diet that mimics a starvation state with sufficient caloric intake to sustain growth and development. KD is an established treatment for several diseases, and it is currently evaluated in the management of insulin-resistant states, although insulin secretion after a classic ketogenic meal has never been investigated. We measured the insulin secretion to a ketogenic meal in 12 healthy subjects (50% females, age range 19–31 years, BMI range 19.7–24.7 kg/m^2^) after cross-over administrations of a Mediterranean meal and a ketogenic meal both satisfying ~40% of an individual’s total energy requirement, in random order and separated by a 7-day washout period. Venous blood was sampled at baseline and at 10, 20, 30, 45, 60, 90, 120, and 180 min to measure glucose, insulin, and *C*-peptide concentrations. Insulin secretion was calculated from *C*-peptide deconvolution and normalized to the estimated body surface area. Glucose, insulin concentrations, and insulin secretory rate were markedly reduced after the ketogenic meal with respect to the Mediterranean meal: glucose AUC in the first OGTT hour −643 mg × dL^−1^ × min^−1^, 95% CI −1134, −152, *p* = 0.015; total insulin concentration −44,943 pmol/L, 95% CI −59,181, −3706, *p* < 0.001; peak rate of insulin secretion −535 pmol × min^−1^ × m^−2^, 95% CI −763, −308, *p* < 0.001. We have shown that a ketogenic meal is disposed of with only a minimal insulin secretory response compared to a Mediterranean meal. This finding may be of interest to patients with insulin resistance and or insulin secretory defects.

## 1. Introduction

The ketogenic diet is a dietary regimen providing very low carbohydrate, high fat, and modest protein, that has an established role in the treatment of drug-resistant epilepsy [1], glucose transporter type 1 deficiency syndrome [2,3,4,5], and other neurologic diseases [6], well tolerated and safe [3,7] also over ten years of continuous application [8,9].

Low carbohydrate and ketogenic diets have become increasingly popular in the treatment of metabolic syndrome, obesity, and type 2 diabetes and various meta-analyses have shown their usefulness [10,11,12], although not conclusively [13].

International consensus establishes carbohydrates as the base of the food pyramid for healthy nutrition. Nevertheless, several studies have shown that an abundant intake of starchy foods and sugars, the main source of energy in Western diets including the Mediterranean Diet, may promote excessive insulin responses [14] with negative health consequences. An excessive ß-cell secretory activity may independently cause weight gain and insulin resistance [15,16,17,18,19,20]. Therefore, it has been hypothesized that diet-induced hyperinsulinemia could be the cause of insulin resistance [21,22], inflammation [23] vasoconstriction [24], and atherogenesis [25] that increase the cardiometabolic risk.

In contrast, ketosis-inducing diets seem to reduce insulin resistance improving glucose and insulin levels, by requiring less insulin to be disposed of, suggesting being particularly useful in patients with insulin resistance triggered or maintained by hyperinsulinemia as well as in those with insulin secretory defects that prevent a normal glucose tolerance. Moreover, ketogenic diets seem to promote a non-atherogenic lipid profile and reduce blood pressure, particularly when associated with weight loss.

Interestingly, ketogenic dietary interventions with ad libitum caloric intake or only moderate caloric restriction (trials reviewed in [26]), produce strong reductions in fasting insulinemia, HOMA-index, and postprandial insulin responses [27] that are disproportionate to the modest differences in weight loss compared to control diets. Therefore, the reduction in insulin concentrations and the improvement in insulin resistance appear to be a direct consequence of this dietary regimen and not mediated by weight loss. The above-mentioned trials [26] showed that the ketogenic diet reduces both parameters in a few weeks but did not ascertain the temporal primacy of one of them. Therefore, it is still unclear whether a ketogenic diet primarily affects insulin secretion and then insulin sensitivity or the reverse.

A reasonable hypothesis is that each of the ketogenic meals in this dietary plan essentially produces an almost flat insulin-secretory response thanks to the very limited amount of carbohydrates provided, despite the presence of proteins and lipids that are known to stimulate insulin secretion, even prior to that ketosis is established and independently from weight loss. Support for this hypothesis would require the exact knowledge of the amount of insulin that is secreted after a ketogenic meal in comparison to an isocaloric Mediterranean meal, but this is not available.

The quantification of insulin required to metabolize a certain meal type cannot be directly obtained by the observation of circulating peripheral insulin concentration profiles, because insulin extraction dynamically changes during the meal as insulin receptors are saturated [28]. However, the quantification of insulin secretion and insulin extraction can be derived by modeling the profile of *C*-peptide, co-secreted with insulin but unaffected by first-pass hepatic extraction [29].

The aim of this study was therefore to measure the insulin secretory response to a typical ketogenic meal providing ~40% of individual energy needs and to compare it to the response to an isocaloric Mediterranean meal in healthy subjects, by modeling the circulating glucose, insulin, and *C*-peptide profiles.

## 2. Materials and Methods

### 2.1. Subjects

This study was conducted at the International Center for the Assessment of Nutritional Status (ICANS), University of Milan (Italy). Twelve healthy subjects (50% females), adults with an age range of 19–31 years, and with a normal weight (BMI range 19.7–24.7 kg/m^2^), were recruited on a voluntary basis among students at the University of Milan. Exclusion criteria were the following: overweight or obese; the presence of diseases causing significant impairment of nutritional status (i.e., Crohn’s disease, neoplasia, end-stage renal failure, cirrhosis, congestive heart failure, and chronic infection); endocrine diseases (i.e., hyper-hypothyroidism and diabetes mellitus); consumption of medications affecting endocrine function within the previous two months; recent (<1 month) occurrence of acute illness or injury; elite athleticism. The characteristics of the study subjects are reported in Table 1. This study was conducted according to the guidelines laid down in the Declaration of Helsinki. Approval was obtained by the Institutional Ethical Committee (n. 32/17, 19 September 2017) and informed consent was signed by all subjects.

### 2.2. Experimental Protocol

In a randomized, cross-over study, the subjects received mixed standardized meals of different compositions on two different days spaced apart by a washout period of 7 days. The meals were consumed at 9 o’clock in the morning after having fasted for at least 12 h. Each meal satisfied ~40% of an individual’s total energy requirement, estimated by multiplying predicted resting energy expenditure [30] by the corresponding physical activity level [31]. Each subject consumed two meals of identical energy content but differing in macronutrient composition:

Mediterranean meal: consisted of a sandwich of white bread, ham, oil, and tomato. Of the energy provided (39.1 ± 1.3% of TEE), 55.4 ± 1.1% was derived from carbohydrates (136.4 ± 16.3 g), 27.0 ± 0.8% from lipids, and 17.6 ± 1.0% from protein; the content of dietary fiber was 7.1 ± 1.0 g (7.4 ± 0.3 g/1000 kcal).

Ketogenic meal: consisted of mascarpone cheese, hazelnuts, and cocoa powder with a ketogenic ratio (the ratio of the fat amount in grams to the combined protein and carbohydrate amount in grams) of 4:1 (mean 3.95, range 3.78–4.08).

As previously described [3,32], of the energy provided (39.1 ± 1.0% of TEE), 3.4 ± 0.2% was derived from carbohydrates (7.0 ± 1.1 g), 89.5 ± 0.2% from lipids, and 7.1 ± 0.3% from protein; the content of dietary fiber was 2.8 ± 0.4 g (2.9 ± 0.1 g/1000 kcal). The meal had a ketogenic ratio of 4:1 (4 g of lipids for every gram of carbohydrate and protein).

The evening before each experiment, the subjects were asked to consume a standardized dinner consisting of rice or pasta dressed with oil and/or Parmesan cheese and/or tomato sauce, meat or fish, vegetables seasoned with olive oil, bread, and fresh fruit. Water was the only beverage allowed.

On the day of the test, the subjects arrived at the laboratory at 8 o’clock in the morning: they were seated in a comfortable room and intravenous catheters were placed into an antecubital vein. Venous blood samples were obtained at baseline and at 10, 20, 30, 45, 60, 90, 120, and 180 min after test meal consumption in order to assess plasma glucose, insulin, and *C*-peptide. Further samples were collected at 240, 300, and 360 min after the ketogenic meal to account for the slower kinetics of gastric emptying, fat digestion, absorption, and metabolism. Blood samples were immediately centrifuged, and plasma was stored at −80 °C until laboratory analyses. Finally, we asked all subjects to fill in a Checklist of Medication Side Effects within 24 h after the experiments, to detect eventual adverse effects of the meals.

### 2.3. Laboratory Analyses

We measured circulating levels of glucose, insulin, and *C*-peptide at baseline and every 10 min in the first half hour, and every 30 min thereafter. All parameters were assayed by a commercial kit (Roche Diagnostics) with Cobas Integra 400 Plus and Cobas 411 (Roche Diagnostic).

### 2.4. Analysis and Modelling of Meal

Insulin secretion (pmol × min^−1^ × m^−2^): pancreatic insulin secretion, calculated from *C*-peptide deconvolution and normalized to estimated body surface area.

Basal insulin secretion (pmol × min^−1^ × m^−2^): insulin secretion before the start of the meal.

Total insulin secretion (nmol × m^−2^): integral of insulin secretion during the whole meal period.

The parameters of the model were estimated from glucose and *C*-peptide concentrations by regularized least squares, as previously described [29]. Regularization involves the choice of smoothing factors that were selected to obtain glucose and *C*-peptide model residuals with SDs close to the expected measurement error (~1% for glucose and ~4% for *C*-peptide). Insulin secretion rates were calculated from the model every 5 min. The integral of insulin secretion during the meal was denoted as total insulin output.

Incremental insulin secretion: was calculated as total insulin secretion − basal insulin secretion × duration of the study.

Insulin clearance: was calculated in the fasting state as the ratio between fasting insulin secretion and fasting insulin concentration and during the meal as the ratio between the integral of insulin secretion and that of insulin concentration.

Positive areas under the meal curve (AUC) were calculated by trapezoidal integration over the entire meal, only considering values above the baseline.

### 2.5. Statistical Analysis

Statistical analysis was carried out using R version 4.2.1 [33]. Subjects’ characteristics are presented as a median and interquartile range, while results are presented as mean and standard error. To examine the influence of OGTT time and meal type on glucose, insulin, *C*-peptide, and insulin secretion rate, we used the two-way analysis of variance (ANOVA). Fisher’s least significant difference (LSD) procedure was used for post-hoc multiple comparisons of means. A *p*-value < 0.05 was considered statistically significant.

## 3. Results

Means of OGTT parameters and modeled parameters by OGTT time, and results from two-way ANOVA are reported in Table 2.

### 3.1. Glucose, Insulin, and C-Peptide

**Figure 1:**
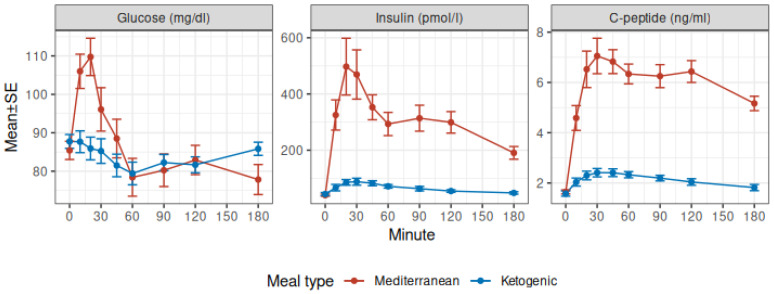
Glucose, insulin, and *C*-peptide profiles by meal type.

The time courses of glucose, insulin, and *C*-peptide concentrations after the Mediterranean and Ketogenic meals are shown in Figure 1.

Fasting state. Fasting plasma glucose, insulin, and *C*-peptide were not clinically different between the test meal studies (Fisher LSD *p*-values 0.64, 0.94, and 0.90, respectively).

Glucose. After the Mediterranean meal, glucose concentration increased and reached an incremental peak of 24 ± 3 mg/dL at 20 min, then decreased and returned to baseline at 60 min (Fisher LSD *p* = 0.17). After both meals, glucose concentration decreased significantly at 60 min (Fisher LSD *p* = 0.04) with a decremental nadir of −8 mg/dL, 95% CI −15, −1 and then returned to baseline at 90 min (Fisher LSD *p* = 0.14). Compared to the Mediterranean meal, glucose concentrations after the ketogenic meal were significantly lower between 10 and 30 min (Fisher LSD *p*-values: 10 min *p* < 0.001, 20 min *p* < 0.001, 30 min *p* = 0.04). The mean glucose concentration during the whole study was not different (Mediterranean − ketogenic: 62 (95% CI 43, 80), *p* < 0.001), but the mean glucose concentration was higher in the first 60 min after the Mediterranean meal (Mediterranean − ketogenic: 11 (95% CI 2.5, 19), *p* = 0.015).

Insulin. After the Mediterranean meal, insulin concentration increased and reached an incremental peak of 457 ± 99 pmol/L at 20 min, which was 12.4 ± 1.9 fold the basal concentration, then it decreased but remained elevated until the end of the study. After the ketogenic meal, insulin concentration increased significantly to reach an incremental peak of 44 ± 10 pmol/L at 30 min, which was 2.2 ± 0.4 fold the basal concentration, then it slowly decreased and returned to the basal concentration at 180 min (Fisher LSD *p* = 0.06). Compared to the Mediterranean meal, insulin concentrations after the ketogenic meal was significantly lower at all time points after baseline (all Fisher LSD *p*-values < 0.001, except at 180 min *p* = 0.01). The mean insulin concentration during the study was also lower (Mediterranean − ketogenic: 250 (95% CI 171, 329), *p* < 0.001).

C-peptide. After the Mediterranean meal, *C*-peptide concentration increased and reached an incremental peak of 5.41 ± 0.65 ng/mL at 30 min, which was 4.24 ± 0.28 fold the basal concentration, then it decreased but remained elevated for the whole of the study. After the ketogenic meal, *C*-peptide concentration increased significantly to reach an incremental peak of 0.83 ± 0.14 ng/mL at 30 min, which was 1.56 ± 0.11 fold the basal concentration, then it slowly decreased but never returned to the basal concentration during the duration of the study. Compared to the Mediterranean meal, incremental *C*-peptide concentrations after the ketogenic meal were significantly lower at all time points after baseline (all Fisher LSD *p*-values < 0.001). The mean *C*-peptide concentration during the study was also lower (Mediterranean − ketogenic: 4.0 (95% CI 3.2, 4.8), *p* < 0.001).

### 3.2. Modeled Insulin Secretion and Insulin Clearance

**Figure 2:**
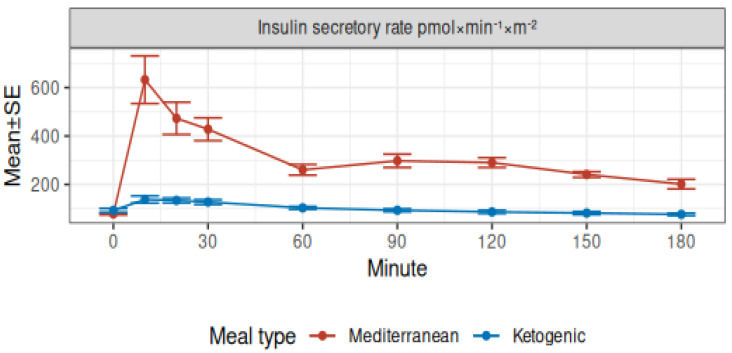
Insulin secretory rate profile by meal type.

The time course of the insulin secretory rate is shown in Figure 2.

Fasting state. Fasting insulin secretion and clearance were consistent with previous reports in healthy subjects [34,35] and were not clinically different between the test meal studies (Mediterranean − ketogenic: insulin secretion −14 (95% CI −32, 2.7), *p* = 0.091; insulin clearance −0.36 (95% CI −0.95, 0.22), *p* = 0.2).

Insulin secretion. After the Mediterranean meal, insulin secretion increased and reached an incremental peak of 554 ± 97 pmol × min^−1^ × m^−2^ at 10 min, which was 8.1 ± 1.3-fold the basal concentration, then it decreased but remained elevated for the whole study. After the ketogenic meal, insulin secretion increased significantly to reach an incremental peak of 45 ± 8 pmol × min^−1^ × m^−2^ at 10 min, which was 1.5 ± 0.1 fold the basal concentration, then it slowly decreased and returned to the basal concentration at 60 min (mean compared to 0: *p* = 0.3). Compared to the Mediterranean meal, incremental insulin concentrations after the ketogenic meal were significantly lower at all time points after baseline (all Fisher LSD *p*-values < 0.001). The mean insulin secretion during the study was also lower (Mediterranean − ketogenic: 238 (95% CI 201, 276), *p* < 0.001).

Table 3 reports total insulin secretion and the mean insulin clearance in the 3 h after the meals. Table 3 shows that the basal rate of insulin secretion increased to a peak rate that was 8.9 ± 1.2 folds the basal after the Mediterranean meal, whereas the peak rate after the ketogenic meal was only 1.8 ± 0.1 folds the basal. During the 3-h study, the incrementally secreted insulin was 17 ± 2 times larger after MED than the KETO meal, which would correspond to approximately 11.9 ± 0.8 IU of insulin vs 0.8 ± 0.1 IU of insulin.

Finally, insulin clearance was reduced during the test when compared to basal values (0.54 ± 0.04 times during the Mediterranean meal, 0.70 ± 0.12 times during the ketogenic meal), but the effect was more pronounced after the Mediterranean meal (total/basal insulin clearance was 0.16 ± 0.12 times lower in the Mediterranean meal).

## 4. Discussion

The quantification of insulin required to metabolize a certain meal type is basic information that may help to predict or to explain the prandial response of subjects affected by insulin secretory defects or by insulin resistance, and to rationally allocate patients to personalized dietary treatments. We found that a Mediterranean meal accounting for 40% of daily dietary intake, requires, for its metabolism, the production of 7.8 ± 0.8 times the amount of insulin compared to fasting values, temporarily spiking the insulin secretory rate to 8.9 ± 1.2-fold the basal values. This marked insulin response is further amplified in the peripheral tissues by a reduced insulin clearance after the meal (OGTT insulin clearance was 0.54 ± 0.04 times the basal values after the Mediterranean meal), which is largely due to first-pass liver uptake. In sharp contrast, a ketogenic meal accounting for the same caloric amount requires a much lesser amount of insulin, increased by a small fraction of that produced post-absorptively, and smaller changes in insulin clearance.

Several points need to be underscored to place our results in the proper context. First, we describe here the insulin secretory responses of healthy subjects. We do not anticipate that the response to the ketogenic meal could be amplified or further reduced in subjects with obesity, metabolic syndrome, or type 2 diabetes, but after a Mediterranean meal, we can imagine an increased insulin secretion in insulin-resistant subjects and the opposite in subjects with insulin secretory defects, both situations potentially leading to hyperglycemia. Both situations could benefit from a ketogenic meal, as reduced prandial insulin secretion and glucose concentration would translate into better insulin sensitivity, a hypothesis that deserves further investigation.

Second, we tested an isocaloric meal, that is not intended to be placed in the context of a hypocaloric diet. This experiment allowed us to quantify the physiologic effect of a ketogenic meal independent of caloric restriction. The information derived can be translated into the context of a maintenance diet, but, obviously, insulin secretion is expected to be reduced if smaller size meals are provided to pursue weight loss.

Third, we tested the effect of a single meal on subjects that were following a western diet. From our data, we cannot predict the insulin secretory responses of subjects in chronic ketosis on a ketogenic diet. Nonetheless, the insulin concentrations profiles reported by [27] were flattened after ketogenic meals in comparison to control meals in a way very similar to the experiments described here. Taken together, our data suggest that the disposal of a ketogenic meal requires an amount of insulin secretion that is smaller compared to a Mediterranean meal, independently from caloric reduction, weight reduction, and prevailing ketosis.

The role of a ketogenic diet in the management of obesity, metabolic syndrome, type 2 diabetes, and other insulin-resistant conditions has been mainly exploited in the context of weight loss programs in which the generation of ketone bodies may reduce appetite, promote satiety and provide the brain with a fuel alternative to glucose [36]. Our study underscores another aspect that can be valuable in the treatment of the same diseases, i.e., that ketogenic meals (even single meals) can satisfy individual energy requirements without significant amounts of insulin secretion. This is an essential feature of ketogenic meals that would help to reduce hyperinsulinemia-driven insulin resistance [22] and would help patients with limited insulin secretory capability to metabolize their food intake without developing significant hyperglycemia. In practical terms, this is reflected in the number of international units of insulin that were required in our study to manage the number of carbohydrates contained in the Mediterranean and ketogenic meal, which are slightly less than the amounts that are suggested as a starting point for carbohydrate counting in type I diabetes [37], probably due to the difference in efficiency between exogenous and endogenous insulin.

Notice that the hyperinsulinemic action of the Mediterranean meal is not limited to first-phase insulin secretion, but is also presented in the second phase, where we see an elevation of insulin concentrations despite normalization of glucose levels approximately at the end of the first hour of OGTT. This may be due to the overall meal size and mediated by incretin action. [35] highlighted how two meals of different sizes (in their study 260 kcal and 520 kcal) can produce quite different insulin profiles while maintaining similar glucose excursion after the meal: in the larger meal, they show an equally fast insulin response in the first phase, coupled with a prolonged and heightened insulin secretion in the second phase. They show how this difference is mediated by differences in Glucagon-like peptide-1 (GLP-1) and glucose-dependent insulinotropic polypeptide (GIP) secretion that cause insulin elevation despite normalization of blood glucose. In our study, the more dramatic effect may be due to the size of the even larger meals (951 (840, 1037) kcal for the Mediterranean meal and 950 (848, 1019) kcal for the ketogenic meal), and may also explain the insulin response to the ketogenic meal in absence of a significant carbohydrate intake [38].

Insulin clearance by the liver is a long-recognized phenomenon that is now being revaluated as a possible factor in diabetes risk. Insulin is secreted in a pulsatile fashion by pancreatic β cells into the portal vein and reaches the liver, where up to 80% of secreted insulin is degraded after receptor-mediated uptake [39]. The evolutionary reasons behind this first passage to the liver are not well understood, but our data reinforce the notion that insulin clearance is a dynamic variable and controllable parameter in the overall regulation of systemic insulin levels [40]: when insulin requirements are higher, such as after eating a high-carbohydrate meal (here the Mediterranean meal), insulin clearance does appear to be suppressed more than after a low-carbohydrate meal (here the ketogenic meal), in order to achieve higher systemic insulin concentrations at equal insulin secretion rates.

This study has several strengths: the cross-over design limited the influence of inter-personal variability on the outcomes; the caloric content of the meals was tailored to individual energy requirements, to more closely mimic the free-living weight-maintenance diet; modeling of pancreatic parameters has highlighted the physiological bases of the differences displayed in insulin response of the two meals. Nonetheless, this study has some limitations: the sample size was small, although reasonably sized to the expected effect size; basal energy requirements were predicted rather than measured.

In conclusion, we have shown that a ketogenic meal is disposed of with only a minimal insulin secretory response compared to a Mediterranean meal. This finding may be of interest to patients with insulin resistance and or insulin secretory defects.

## Figures and Tables

**Table 1:** Subjects’ characteristics.

Characteristic	Overall, *n* = 12 ^1^	Female, *n* = 6 ^1^	Male, *n* = 6 ^1^
Age (years)	24.1 (23.1, 25.2)	23.7 (23.3, 24.6)	24.7 (23.2, 25.7)
Body mass index (kg/m^2^)	21.5 (20.4, 23.4)	21.8 (20.3, 23.0)	21.5 (20.7, 23.7)
Waist circumference (cm)	73.9 (72.7, 78.4)	72.6 (72.4, 73.5)	76.7 (74.3, 78.8)
Fat mass fraction (as %)	20.7 (16.4, 30.0)	30.1 (28.6, 33.9)	15.9 (12.3, 17.4)
Total energy expenditure (kcal)	2393 (2164, 2655)	2156 (2139, 2215)	2679 (2555, 2776)

^1^ Median (IQR).

**Table 2:** Means of OGTT parameters and modeled parameters by OGTT time, and results from two-way ANOVA examining the influence of OGTT time and meal type on each parameter.

		OGTT Minutes								Two-Way ANOVA *p*-Values ^2^		
Characteristic	Group	0 ^1^	10 ^1^	20 ^1^	30 ^1^	60 ^1^	90 ^1^	120 ^1^	180 ^1^	Minute	Meal	Interaction
Glucose (mg/dL)										**<0.001**	**0.005**	**<0.001**
	Mediterranean	85 ± 2	106 ± 4	110 ± 5	96 ± 6	78 ± 5	80 ± 4	83 ± 4	78 ± 4			
	Ketogenic	88 ± 2	88 ± 3	86 ± 3	85 ± 3	79 ± 3	82 ± 2	82 ± 2	86 ± 2			
Insulin (pmol/L)										**<0.001**	**<0.001**	**<0.001**
	Mediterranean	40 ± 4	325 ± 54	497 ± 101	469 ± 88	293 ± 41	314 ± 46	299 ± 38	190 ± 23			
	Ketogenic	44 ± 5	67 ± 11	86 ± 10	88 ± 12	71 ± 6	63 ± 8	55 ± 5	48 ± 4			
*C*-peptide (ng/mL)										**<0.001**	**<0.001**	**<0.001**
	Mediterranean	1.65 ± 0.08	4.58 ± 0.49	6.52 ± 0.72	7.05 ± 0.71	6.34 ± 0.39	6.25 ± 0.46	6.43 ± 0.43	5.16 ± 0.29			
	Ketogenic	1.58 ± 0.10	2.04 ± 0.16	2.30 ± 0.17	2.41 ± 0.17	2.33 ± 0.12	2.19 ± 0.11	2.04 ± 0.13	1.82 ± 0.12			
Insulin secretion rate (pmol × min^−1^ × m^−2^)										**<0.001**	**<0.001**	**<0.001**
	Mediterranean	78 ± 4	632 ± 98	473 ± 66	428 ± 47	261 ± 22	298 ± 27	290 ± 20	202 ± 20			
	Ketogenic	93 ± 8	138 ± 15	134 ± 10	128 ± 10	103 ± 6	94 ± 6	87 ± 7	77 ± 5			

^1^ Mean ± SE. ^2^ Bold indicates *p*-values < 0.05.

**Table 3:** Differences between meals in insulin secretion, insulin concentration, and insulin clearance.

Characteristic	Mediterranean, *n* = 12 ^1^	Ketogenic, *n* = 12 ^1^	Difference ^2^	95% CI ^2,3^	*p*-Value ^2^
Basal rate of insulin secretion (pmol × min^−1^ × m^−2^)	78 ± 4	93 ± 8	−14	−32, 2.7	0.091
Peak rate of insulin secretion (pmol × min^−1^ × m^−2^)	697 ± 95	161 ± 14	535	308, 763	**<0.001**
Basal insulin concentration (pmol/L)	40 ± 4	44 ± 5	−3.8	−12, 4.1	0.3
Incremental insulin concentration (pmol/L)	7929 ± 999	668 ± 110	7261	5040, 9482	**<0.001**
Total insulin concentration (pmol/L)	54,764 ± 6378	9821 ± 1103	44,943	30,706, 59,181	**<0.001**
Basal insulin clearance (l × min^−1^ × m^−2^)	2.12 ± 0.18	2.48 ± 0.39	−0.36	−0.95, 0.22	0.2
Insulin clearance during the test (l × min^−1^ × m^−2^)	1.10 ± 0.08	1.39 ± 0.18	−0.30	−0.74, 0.15	0.2

^1^ Mean ± SE. ^2^ Paired *t*-test, bold *p*-values are < 0.05. ^3^ CI = Confidence Interval.

## Data Availability

Data are available upon reasonable request.
